# Intravascular ICG-enhanced NIRF-IVUS imaging to assess progressive atherosclerotic lesions in excised human coronary arteries

**DOI:** 10.1038/s44325-024-00016-8

**Published:** 2024-08-30

**Authors:** Philipp Rauschendorfer, Tobias Lenz, Philipp Nicol, Léa Wild, Alicia Beele, Emina Sabic, Grace Klosterman, Karl-Ludwig Laugwitz, Farouc A. Jaffer, Dimitris Gorpas, Michael Joner, Vasilis Ntziachristos

**Affiliations:** 1 Chair of Biological Imaging at the Central Institute for Translational Cancer Research (TranslaTUM), School of Medicine and Health, Technical University of Munich, Munich, Germany; 2 Institute of Biological and Medical Imaging, Helmholtz Zentrum München, Neuherberg, Germany; 3 DZHK (German Centre for Cardiovascular Research), Partner Site Munich Heart Alliance, Munich, Germany; 4 Klinik für Herz- und Kreislauferkrankungen, Deutsches Herzzentrum München, Technical University Munich, Munich, Germany; 5 Cardiovascular Research Center, Cardiology Division, Massachusetts General Hospital, Harvard Medical School, Boston, MA USA; 6 Munich Institute of Biomedical Engineering (MIBE), Technical University of Munich, Garching b, München Germany

**Keywords:** Cardiovascular diseases, Medical imaging

## Abstract

Indocyanine green (ICG)-enhanced intravascular near-infrared fluorescence (NIRF) imaging enhances the information obtained with intravascular ultrasound (IVUS) by visualizing pathobiological characteristics of atherosclerotic plaques. To advance our understanding of this hybrid method, we aimed to assess the potential of NIRF-IVUS to identify different stages of atheroma progression by characterizing ICG uptake in human pathological specimens. After excision, 15 human coronary specimens from 13 adult patients were ICG-perfused and imaged with NIRF-IVUS. All specimens were then histopathologically and immunohistochemically assessed. NIRF-IVUS imaging revealed colocalization of ICG-deposition to plaque areas of lipid accumulation, endothelial disruption, neovascularization and inflammation. Moreover, ICG concentrations were significantly higher in advanced coronary artery disease stages (*p* < 0.05) and correlated significantly to plaque macrophage burden (*r* = 0.67). Current intravascular methods fail to detect plaque biology. Thus, we demonstrate how human coronary atheroma stage can be assessed based on pathobiological characteristics uniquely captured by ICG-enhanced intravascular NIRF.

## Introduction

Coronary artery disease (CAD) is a global healthcare burden that is associated with 17.8 million deaths annually^1^. Imaging technologies like intravascular ultrasound (IVUS) and intravascular optical coherence tomography (IVOCT) have been regularly used in the clinics to guide percutaneous coronary interventions and improve procedural outcome^2^. Yet, there is no recommendation to use intravascular imaging for the detection of high-risk coronary atherosclerotic lesions, nor to utilize these technologies for clinical decision-making guidance and therapeutic planning in patients with acute and chronic coronary syndrome^3^. A major reason is that morphology-based intravascular imaging modalities (IVUS and IVOCT) are not suitable for detecting pathophysiological features of high-risk coronary lesions, such as high endothelial permeability, lipid accumulation, and inflammation^4^. Intravascular near-infrared fluorescence (NIRF) imaging operating in tandem with IVUS or IVOCT in a hybrid imaging catheter^5–9^ has been proposed for visualization of these pathophysiological markers through imaging fluorescently labeled reporters that can target and reveal underlying biological processes^10^. Complementing biological information from NIRF with anatomical readouts from IVUS or IVOCT promises a more comprehensive characterization of the vascular wall in patients with CAD^10,11^. So far, the NIRF technology, mostly in combination with the FDA-approved NIRF-agent indocyanine green (ICG), has shown promising results in different animal models of atherosclerosis^5,6,9,12–15^ and human carotid endarterectomy specimens^16^, but has never been investigated in human coronary artery disease.

Therefore, the primary aim of this study is to characterize ICG-uptake in human pathological specimen with different stages of CAD at autopsy and to assess the performance of NIRF-IVUS to detect ICG-deposition within the arterial wall. We further aimed to investigate the ability of NIRF-IVUS to detect different concentrations of intraplaque ICG-deposition in order to discriminate different stages of atheroma progression as a function of ICG uptake. Lastly, given the importance of plaque inflammation during progression of coronary atherosclerosis, we specifically intended to develop a tool for quantification of intimal macrophage infiltration by ICG-enhanced NIRF-IVUS.

## Results

### NIRF-IVUS imaging detected high ICG signals in plaque areas with advanced stages of atheroma and low ICG signals in early-stage intimal lesions of human coronary arteries

**Fig. 1:**
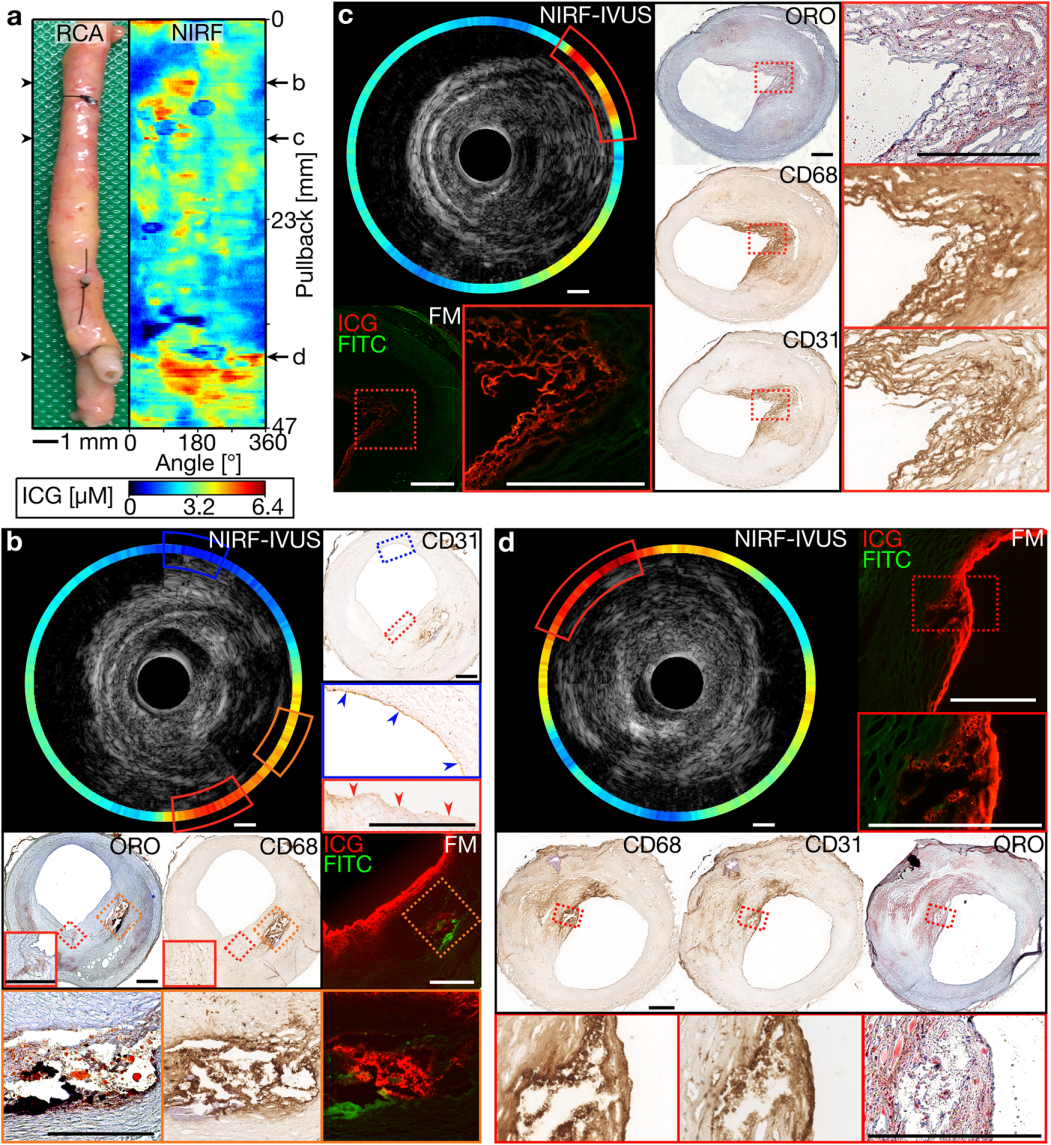
NIRF-IVUS imaging detected high ICG-concentrations spatially related to plaque areas with accumulated lipids, infiltrated macrophages and endothelial disruption. **a** Picture and corresponding NIRF map of an ICG-perfused RCA with representative pullback locations co-registered to tissue cryosections for histological assessment and confirmation of ICG-accumulation by FM (**b**–**d**); (**b**) Cross-sectional NIRF-IVUS image showcasing high ICG-concentrations (NIRF-IVUS- red and orange box) co-localized to plaque regions with accumulated lipids (ORO – orange and red box), infiltrated macrophages (CD68 – orange and red box) and absent endothelial cells (CD31 - red box; red arrows indicating disrupted endothelium). Low ICG-concentration (NIRF-IVUS - blue box) found in an area of intimal thickening with present endothelial cells (CD31 - blue box; blue arrows indicating undisrupted endothelium); (**c**) cross-sectional NIRF-IVUS image showcasing high ICG-concentrations (NIRF-IVUS - red box) co-localized to a plaque region with accumulated lipids (ORO - red box), macrophages infiltrated into the surface area (CD68 - red box) and neovascularization (CD31 - red box); (**d**) Cross-sectional NIRF-IVUS image showcasing high ICG-concentrations (NIRF-IVUS - red box) spatially related to a superficial plaque area with accumulated lipids (ORO - red box) and infiltrated macrophages (CD68 - red box). In all panels: FITC Fluorescein channel for autofluorescence; FM fluorescence microscopy, ICG indocyanine green, IVUS intravascular ultrasound, NIRF near-infrared fluorescence, ORO oil red O, RCA right coronary artery; Scale bars: 500 µm.

Figure 1 shows a right coronary artery (RCA) as imaged with NIRF-IVUS. Figure 1a displays the imaged segment of the RCA and the corresponding distance-corrected NIRF map, indicating representative pullback locations, co-registered to consecutive histopathological tissue sections (Fig. 1b–d). High ICG-concentration was detected in a cross-sectional NIRF-IVUS frame (NIRF-IVUS - red and orange box, Fig. 1b) co-localized to plaque regions with lipid accumulation (ORO - orange and red box, Fig. 1b), infiltration of macrophages (CD68 -orange and red box, Fig. 1b) and absent endothelial cells indicating endothelial disruption (CD31 - red box, Fig. 1b). ICG-accumulation was confirmed in the intimal and deeper plaque regions (FM—orange box, Fig. 1b) by fluorescence microscopy (FM). In contrast, low ICG-concentration (NIRF-IVUS - blue box, Fig. 1b) was detected in an area with undisrupted (healthy) endothelium (CD31 - blue box, Fig. 1b). Similar patterns of co-localization of ICG to sites of lipid accumulation (ORO; Fig. 1c-d, Fig. S1b, c and S2b–d), macrophage infiltration (CD68, Fig. 1c, d, Fig. S1b, c and S2c, d) and endothelial disruption (CD31, Fig. S2b, c), as opposed to early-stage intimal lesions (Fig. S1d) with intact CD31+ endothelium (CD31, Fig. S2b, c), were observed in various plaques found within the same RCA segment and other coronary segments. Low ICG-uptake, in regions of only early-stage intimal lesions, was further confirmed when imaging a left internal mammary artery (LIMA) bypass-graft, which showed only minor pathological changes on histological assessment throughout the entire artery segment (MP, CD68 and αSMA; Fig. S3b–e). Locally increased ICG-concentrations (NIRF-IVUS – yellow and red boxes) were only detected in tissue areas with signs of endothelial damage extending to the intimal layer, as indicated by co-registered IVOCT (IVOCT - yellow and red boxes, Fig. S3b,e), most likely representing post-mortem tissue damage.

NIRF-IVUS imaging also revealed high ICG-concentrations (NIRF-IVUS - red box, Fig. S1b and S2d; orange box, Fig. S2c) in deeper plaque areas at sites of neovascularization (CD31; Fig. S1b and S2c, d). In some cases, single neo-vessels were co-registered to local ICG hotspots in FM (indicated by * in FM and CD31 - red boxes; Fig. S1b and S2d). Finally, NIRF-IVUS of a stented left anterior descending artery segment (Fig. S4) detected high ICG uptake at the distal stent edge where histology revealed an uncovered fibroatheroma (NIRF-IVUS - red box, Fig. S4b). Stented vessel segments with underlying fibroatheroma fully covered by healthy neo-endothelium showed only minor NIRF-signal (NIRF-IVUS, Fig. S4c-e).

### Intravascular NIRF-IVUS detects an increase in ICG-uptake with progression of atherosclerotic disease burden

**Fig. 2:**
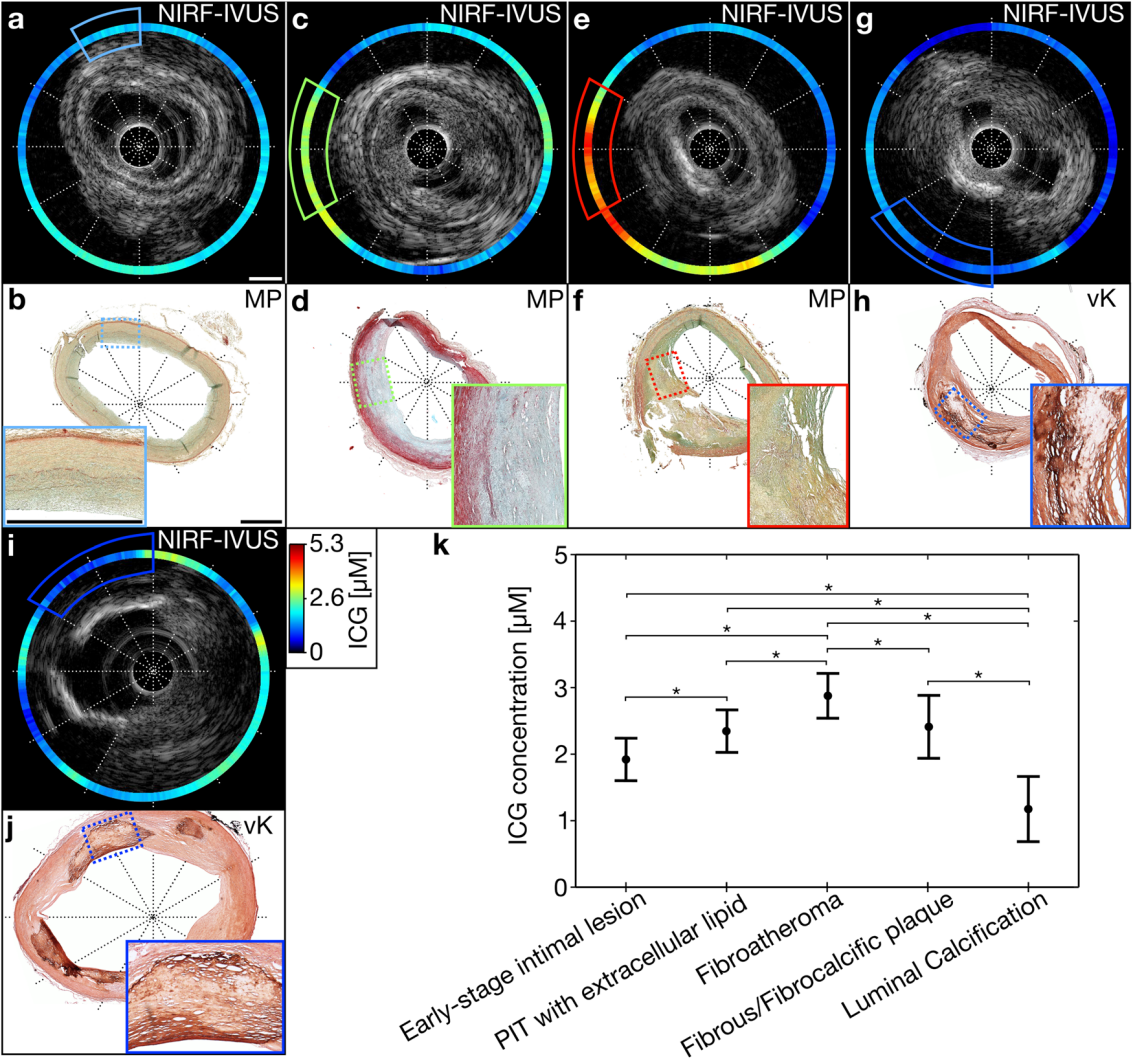
ICG-concentration quantified by NIRF-IVUS depends significantly on the pathological classification of coronary tissue evaluated per 30° sector. Exemplary NIRF-IVUS frames with co-registered histological tissue sections embedded in paraffin and stained with MP and vK indicating 30° tissue segments classified as (**a**, **b**) early-stage intimal lesions, (**c**, **d**) PIT with extracellular lipid, (**e**, **f**) fibroatheroma, (**g**, **h**) fibrous or fibrocalcific plaque, (**i**, **j**) luminal calcification; (**k**) Detected ICG-concentration averaged per 30° sector in various pathological classifications of the tissue. *Significant differences *p* < 0.05; black dots represent the estimated means; whiskers indicate 95% confidence intervals; In all panels: ICG indocyanine green, IVUS intravascular ultrasound, MP Movat Pentachrome, NIRF near-infrared fluorescence, PIT pathological intimal thickening, vK von Kossa. Scale bars: 1 mm.

We compared ICG-concentrations as quantified by intravascular NIRF-IVUS with pathological classification of different coronary atherosclerosis stages per 30° sector for all coronary arteries (Fig. 2). We show representative NIRF-IVUS frames with co-registered histology sections which were paraffin-embedded and stained with Movat’s Pentachrome (MP) and von Kossa (vK) (Fig. 2a–j). We identified sectors as early-stage intimal lesions (Fig. 2a, b; Light blue box), pathological intimal thickening (PIT) with extracellular lipid (Fig. 2c, d; Green box), fibroatheroma (Fig. 2e, f; Red box), fibrous/fibrocalcific plaques (Fig. 2g, h; Blue box) and luminal calcified regions (Fig. 2i–j; Dark blue box). The average ICG-concentration (±s.d.) detected by NIRF-IVUS was 1.9±0.16 µM in early-stage intimal lesion sectors (*n* = 486 sectors), 2.4 ± 0.16 µM in PIT with extracellular lipid sectors (*n* = 349), 2.9±0.17 µM in fibroatheroma sectors (n = 210), 2.4 ± 0.24 µM in fibrous/fibrocalcific plaque sectors (*n* = 23), and 1.2±0.24 µM in sectors with luminal calcification (*n* = 19). We found significantly higher average ICG-concentration in sectors classified as fibroatheroma, with NIRF SNR up to 8.7, compared to early-stage intimal lesions (*p* < 0.001), PIT with extracellular lipid pool (p < 0.001), fibrous/fibrocalcific plaques (*p* = 0.15) and luminal calcification (*p* < 0.001). Furthermore, we found significantly lower average ICG-concentration in sectors classified as early-stage intimal lesions compared to PIT with extracellular lipid (*p* < 0.001) and in luminal calcified areas compared to PIT with extracellular lipid (*p* < 0.001) (Fig. 2k).

### Intravascular NIRF-IVUS signals correlate with the severity of inflammation

**Fig. 3:**
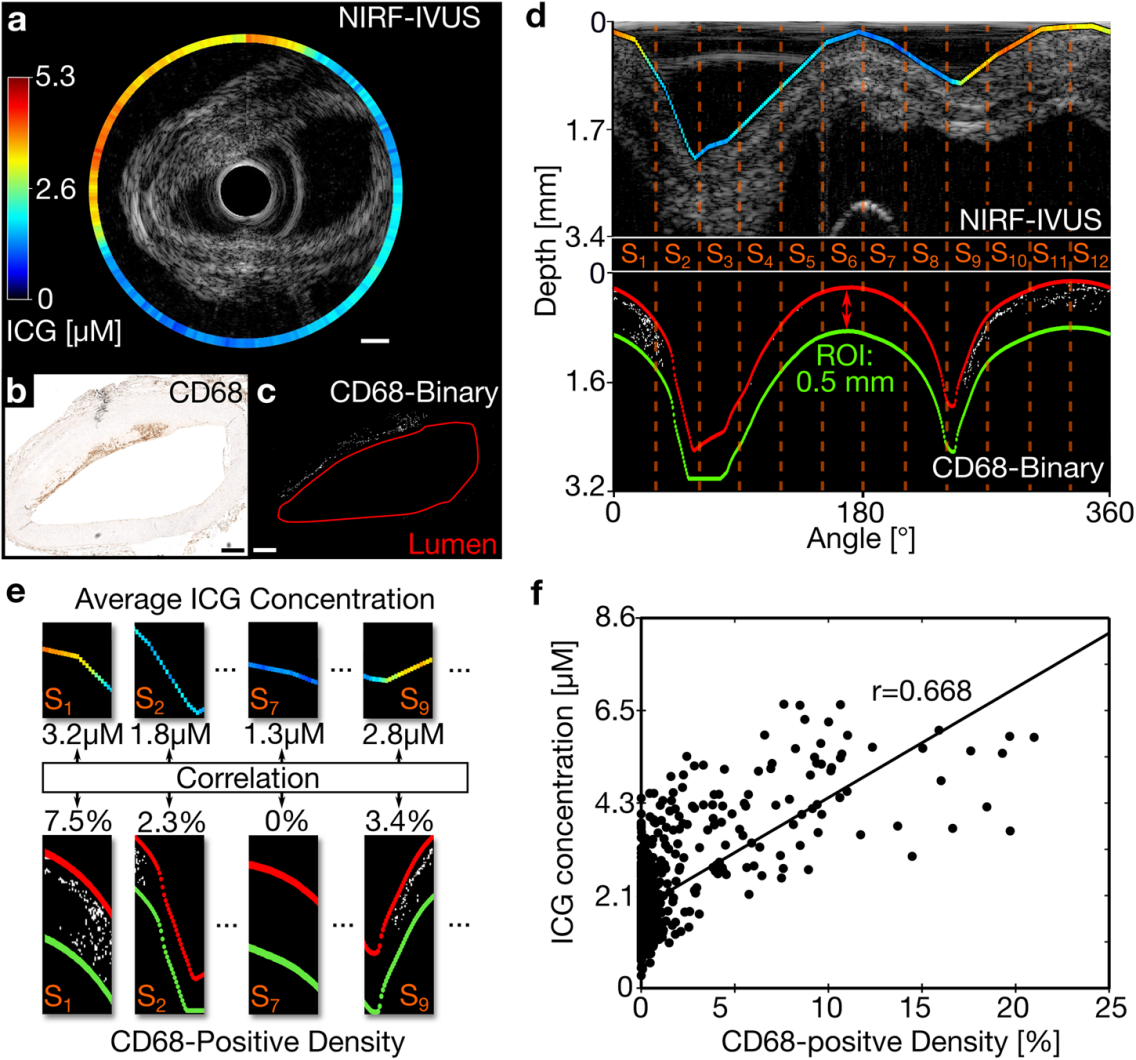
ICG-concentration quantified by NIRF-IVUS strongly correlates to the density of CD68-positive staining indicative of macrophage burden in coronary lesions. **a** Exemplary NIRF-IVUS frame of ICG-perfused coronary artery in cartesian coordinates; (**b**) Co-registered histology section showing CD68-staining in cartesian coordinates; (**c**) Converted binary image of histology section (White pixels indicate CD68 positive staining; lumen outlined in red); (**d**) NIRF data (Projected on IVUS image) and binarized CD68-histology image in polar coordinates showing co-registered 30° sectors (S_1_-S_12_). ROI defined as area between lumen (Red) and 0.5 mm tissue depth (Green); (**e**) Average ICG-concentration correlated to density of CD68-positive binary signal in the ROI per sector; (**f**) Significant positive correlation between quantified ICG-concentration and density of CD68-positive pixels; *n* = 792 and *p* < 0.01; Line of best fit shown in black; In all panels: ICG indocyanine green, IVUS intravascular ultrasound, NIRF near-infrared fluorescence, ROI region of interest; Scale bars: 500 µm.

In order to assess the ability of NIRF-IVUS to quantify inflammation, we correlated ICG-concentrations as quantified by NIRF-IVUS per tissue sector with the intensity of CD68 positive staining. Figure 3a–e shows the implementation of the image processing algorithm (described in the Methods) for a representative NIRF-IVUS frame and corresponding histological cross-section. Using this method, we found a significant and strong positive correlation (Average *r* = 0.668, *p* < 0.01) between the quantified ICG-concentration and calculated density of CD68-positive pixels—measure of the severity of inflammation—for all sectors (*n* = 792) included in this analysis (Fig. 3f). We found a small variation between folds (*n* = 10; Var = 0.0009) in our leave-one-out cross-validation approach, indicating high consistency of the data included in this analysis.

**Fig. 4:**
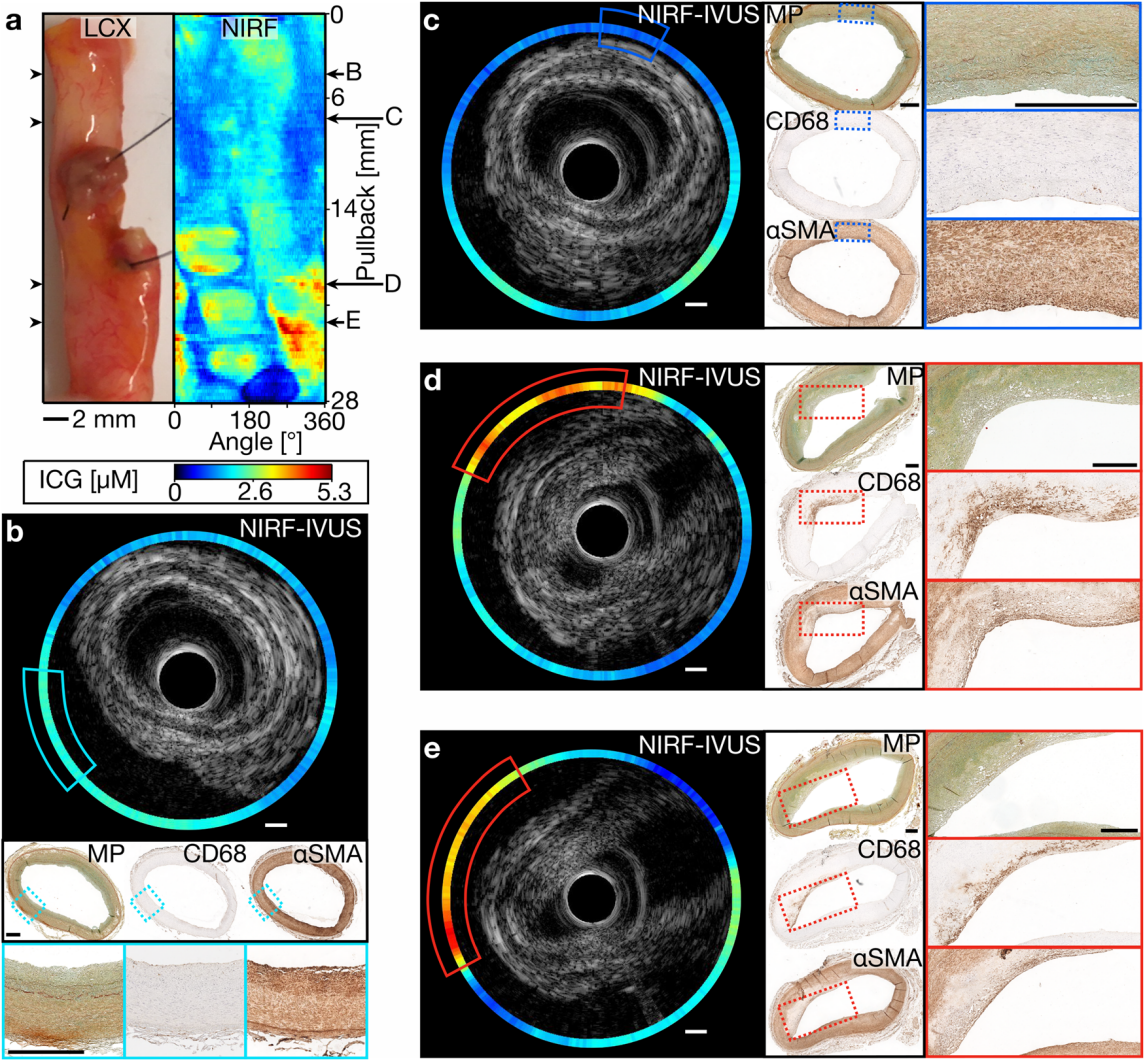
NIRF-IVUS imaging detected low ICG-concentrations in early-stage and high ICG-concentrations in more advanced atherosclerotic lesions. **a** Picture and corresponding NIRF map of an ICG-perfused LCX with representative pullback locations co-registered with consecutive paraffin-embedded tissue sections stained for histological assessment (**b**–**e**). **b**, **c** Cross-sectional NIRF-IVUS images showing low ICG-concentrations in tissue regions of early-stage intimal lesions consistent with validation done by MP, CD68 and αSMA staining; (**d**, **e**) Cross-sectional NIRF-IVUS images showing high ICG-concentrations co-localized to more advanced lesions showing subendothelial foam cell formation as evidenced by the presence of macrophages (Brown signal in CD68 - red boxes) with bubbly cytoplasm, extracellular lipid (MP - red boxes) and a lack of smooth-muscle cells (Lack of brown signal in αSMA - red boxes). In all panels: ICG indocyanine green, IVUS intravascular ultrasound, LCX left circumflex artery, MP movat's pentachrome, NIRF near-infrared fluorescence, αSMA alpha-smooth muscle actin. Scale bars: 500 µm.

Figure 4 shows NIRF-IVUS images of an ICG-perfused coronary artery with different stages of PIT. In accordance with the significant positive correlation between ICG-concentration and severity of inflammation, low ICG-concentrations were detected in tissue sections of early-stage intimal lesions (NIRF-IVUS), without evident macrophage infiltration (CD68, Fig. 4b, c). Figure 4d, e displays NIRF-IVUS images with high ICG-concentrations (NIRF-IVUS - red boxes) co-localized to regions of PIT with extracellular lipid and foam cell infiltration (CD68 - red boxes).

## Discussion

We present herein the first NIRF-IVUS study that investigated co-localization patterns of ICG with pathophysiological characteristics of coronary atherosclerosis in different disease progression stages, using human coronary segments.

Our aim was to provide a foundation for the understanding of ICG-accumulation in early and more advanced disease stages and to test the feasibility of NIRF-IVUS imaging to detect clinically relevant pathobiological features of human coronary atherosclerosis. In this regard, the most salient findings were:

(i) the co-localization of NIRF-detected ICG to pathological markers of coronary atheroma like inflammation, lipid accumulation, signs of endothelial disruption, and neovascularization;
(ii) the increase of ICG-concentration, quantified on the basis of NIRF-intensity, with atherosclerotic plaque progression and decrease in calcified lesions;
(iii) the strong positive correlation between intimal macrophage infiltration and local ICG-concentration.

In our study, we found ICG-deposition in human atherosclerotic lesions, localized in regions with a discontinuous endothelial cell layer indicating increased endothelial permeability, accumulation of lipids and inflammatory cells, and signs of neovascularization. The co-registration of NIRF-IVUS cross-sections to corresponding histology, also showed, that ex vivo NIRF-IVUS detects localized ICG-deposition within atherosclerotic lesions. This is broadly in line with the results of previous animal studies, in which administration of ICG enabled NIRF-(IVUS/IVOCT) to identify areas of angioplasty-induced vascular injury and inflamed lipid-rich plaques^5,9,17^. Until now, the only investigation of ICG-enhanced NIRF imaging in the context of human atherosclerosis is the study by Verjans et al. on excised plaques retrieved from carotid endarterectomy^16^. In that study, five patients were injected with ICG prior to carotid endarterectomy and FM and ex vivo NIRF-IVOCT imaging detected ICG diffusion into plaques through areas of endothelial disruption (leakage) and ICG-accumulation in the presence of macrophages, lipids, and intraplaque hemorrhage. These histopathological features, correlated to the ICG-uptake, are known key contributors to both initiation of coronary atherosclerosis and progression from early lesions to advanced high-risk plaques^18–21^. In our study, we also observed colocalization of ICG to areas of intraplaque neovascularization as detected by FM and CD31-immunostaining. Neovascularization is known to drive plaque progression towards late fibroatheroma through an increase in plaque permeability caused by immature endothelial cells with impaired barrier function lining the newly sprouting neovessels, ultimately resulting in intraplaque hemorrhage, the hallmark of late fibroatheroma^19^. Our data suggests that intraplaque neovessels may be a transport mechanism for ICG in addition to endoluminal diffusion, explaining ICG-accumulation in deeper regions of coronary atheroma. The ability to detect localized fluorescent tracer deposition within atherosclerotic lesions highlights the potential of NIRF-IVUS to meet the clinical need for detection of the pathobiological processes that define the risk for progression and vulnerability of atherosclerotic plaques.

We, further, investigated the relationship between NIRF-quantified ICG-concentrations within plaques and progression of atherosclerosis from early intimal lesions to fibroatheroma. We found a significantly increased average ICG-concentration in fibroatheroma (2.9 µM), compared to PIT with extracellular lipid pool (2.4 µM) and early-stage intimal lesions (1.9 µM). The specificity of NIRF-IVUS regarding the detection of atherosclerotic lesions according to intralesional ICG-deposition was further strengthened when a LIMA bypass was imaged. Histopathology showed only minor atherosclerotic changes in the internal mammary artery segment, commonly known for its unique biological properties that protect it from atherosclerosis and make it a superior choice of graft for coronary artery bypass surgery^22,23^. The detected ICG-concentration, averaged over the entire pullback length (38 mm) of this artery, was 21% lower than concentrations found in tissue classified as PIT with extracellular lipid pool and 35% lower than in tissue classified as fibroatheroma. Additionally, our analysis showed significantly lower average ICG-concentration in calcified luminal regions (1.2 µM), suggesting that macroscopic calcifications potentially blocked or slowed ICG diffusion into the deeper tissue areas. Similarly, we observed a significantly lower average ICG-concentration in fibrous/fibrocalcific plaques (2.4 µM) compared to fibroatheroma (2.9 µM). This decreased plaque permeability for ICG could be the result of similar mechanisms that are responsible for reduced flux of lipidic, inflammatory, and necrotic components commonly observed in calcified plaques, generally regarded as stable lesions^24–26^. NIRF did not detect a significant difference in ICG-concentration between PIT with extracellular lipid pool and fibrous/fibrocalcific plaques. However, co-registered IVUS allowed differentiation between both classifications due to the increased echogenicity of calcified tissue in comparison to non-calcified tissue^27^, which underlines the benefits of a hybrid examination combining biological (NIRF) with anatomical (IVUS or IVOCT) information. Overall, these findings demonstrate the feasibility of NIRF-IVUS to detect progressive atherosclerotic plaque phenotypes, based on increased ICG-deposition at sites of key pathobiological hallmarks of high-risk coronary lesions, with high specificity as indicated by low NIRF signals in early-stage intimal lesions and stable fibrous/fibrocalcific plaques. Therefore, subject to the limitations regarding clinical translation of experimental intravascular imaging technologies, contrast-enhanced near-infrared molecular imaging has the potential to improve the detection of early, clinically inapparent coronary atherosclerotic lesions, with a high risk for progression and to distinguish stable from high-risk lesions with otherwise similar morphological appearance according to established imaging modalities.

Since past studies in the field were limited to the qualitative assessment of co-localization between presence of atheroma-altered pathological markers and detected NIRF signals, we performed additional quantitative histopathological validation focussing on intimal macrophage infiltration as a measure of inflammatory activity and overall atherosclerosis disease stages. The qualitative histology assessment in this study suggests that there may also be a quantifiable correlation between lipid accumulation and NIRF signals. However, ORO staining showed a significant color variance and, thus, requires a much larger sample size for an unbiased quantification with our processing algorithm. We plan a follow-up study where additional data will be acquired to investigate the potential to correlate ICG-concentrations with ORO staining for lipids. Regarding inflammation, our data demonstrated a strong positive correlation between intralesional ICG-concentrations, as quantified by NIRF-IVUS, and macrophage density estimated by histological imaging analysis (*r* = 0.668). This correlation may further strengthen the ability of NIRF-IVUS to serve as an unbiased tool for the detection of relevant pathophysiological risk criteria of human coronary atherosclerosis, such as the accumulation of CD68-positive inflammatory cells.

Our study was not free from limitations. Ex vivo imaging studies fail to replicate some in vivo conditions like blood flow, tracer uptake through phagocytosis or a fully functional endothelial barrier. A dysfunctional endothelial barrier due to post-mortem degradation might have been responsible for occasional unspecific ICG-uptake resulting in increased NIRF imaging background. However, we did not find large areas with artifacts possibly caused by post-mortem damage based on IVOCT imaging (Fig. S5), or areas of endothelial disruption not explained by an underlying atherosclerotic process based on histopathological assessment. Unlike many other conditions, histopathological examination of human coronary arteries is only possible postmortem and coronary endarterectomy is a rarely performed procedure, which precludes an ex vivo imaging study with prior in vivo ICG administration as performed by Verjans et al. featuring carotid endarterectomy specimen^16^. Although our study was based on ex vivo ICG perfusion and imaging of coronary arteries obtained through autopsy, robust NIRF contrast between early and more advanced stages of atherosclerosis was found. We anticipate that contrast will further be improved when investigating ICG-uptake in coronary arteries in vivo. Furthermore, observations in this study are based on co-localization of ICG to pathobiological characteristics of coronary atherosclerosis identified by histopathology. Therefore, conclusions regarding the mechanism behind uptake and intraplaque deposition must be drawn with caution. In a few cases, our intravascular NIRF-IVUS system detected ICG uptake in deeper tissue regions (Maximum depth: 1.2 mm) due to the NIRF sensitivity of the system (Supplemental information 2, Fig. S7d). However, accurate quantification of ICG-concentrations located in deep tissue regions is impossible since intravascular NIRF imaging lacks the depth information needed to correct signal attenuation. Thus, a quantitative analysis should be focused on superficial plaque regions. Combining a superficial, pathobiological characterization by NIRF with deep tissue information, like IVUS plaque burden, promises a comprehensive plaque assessment not feasible with standalone imaging modalities or hybrid fluorescence-IVOCT technologies. In addition, we are planning to increase IVUS center frequency of our hybrid catheter from 40 MHz to 60 MHz to improve image resolution (without compromising penetration depth) for increased visibility of small structural features with clinical significance like stent struts and vessel wall dissection. NIRF-IVUS pullback speed in this study was slow (0.5 mm/s) to sample coronary specimens with high accuracy for precise co-registration with histology. For in vivo applications imaging speed needs to be increased to real-time (>20 FPS and 5 mm/s) to avoid motion artifacts and reduce procedure time. Ultimately, the findings of this work must be evaluated in a large-scale in vivo human imaging study using a clinical dose of ICG. Only then, the prognostic value of quantified NIRF signals can be evaluated in the context of coronary atherosclerosis.

In summary, after ex vivo perfusion, ICG co-localized to plaque areas in human coronary arteries featuring relevant components of human atherosclerosis such as endothelial disruption, neovascularization, a lipid-rich necrotic core, and areas of macrophage infiltration, in concentrations detectable by intravascular NIRF-IVUS. In addition, NIRF-IVUS was able to detect intraplaque ICG-deposition at sites of atherosclerotic lesions and to discriminate different stages of coronary atherosclerosis based on accumulated ICG-concentrations, with significantly higher concentrations found in progressive atherosclerotic lesions as opposed to lower concentrations in early-stage intimal lesions and inactive, calcified lesions. Lastly, ICG-concentrations showed a strong positive correlation to a computed marker of intimal macrophage burden that indicates inflammatory activity.

These findings provide evidence that intravascular ICG-enhanced NIRF-IVUS may serve as a molecular imaging technology to visualize prognostically relevant pathobiological features of atherosclerotic plaques. Molecular imaging of atherosclerotic plaques has the potential to support clinical decision-making by early detection of progressive lesions and discrimination of stable from high-risk plaque phenotypes and to facilitate the implementation of potential future targeted anti-atherosclerotic therapy approaches.

## Methods

### NIRF-IVUS imaging system

**Fig. 5:**
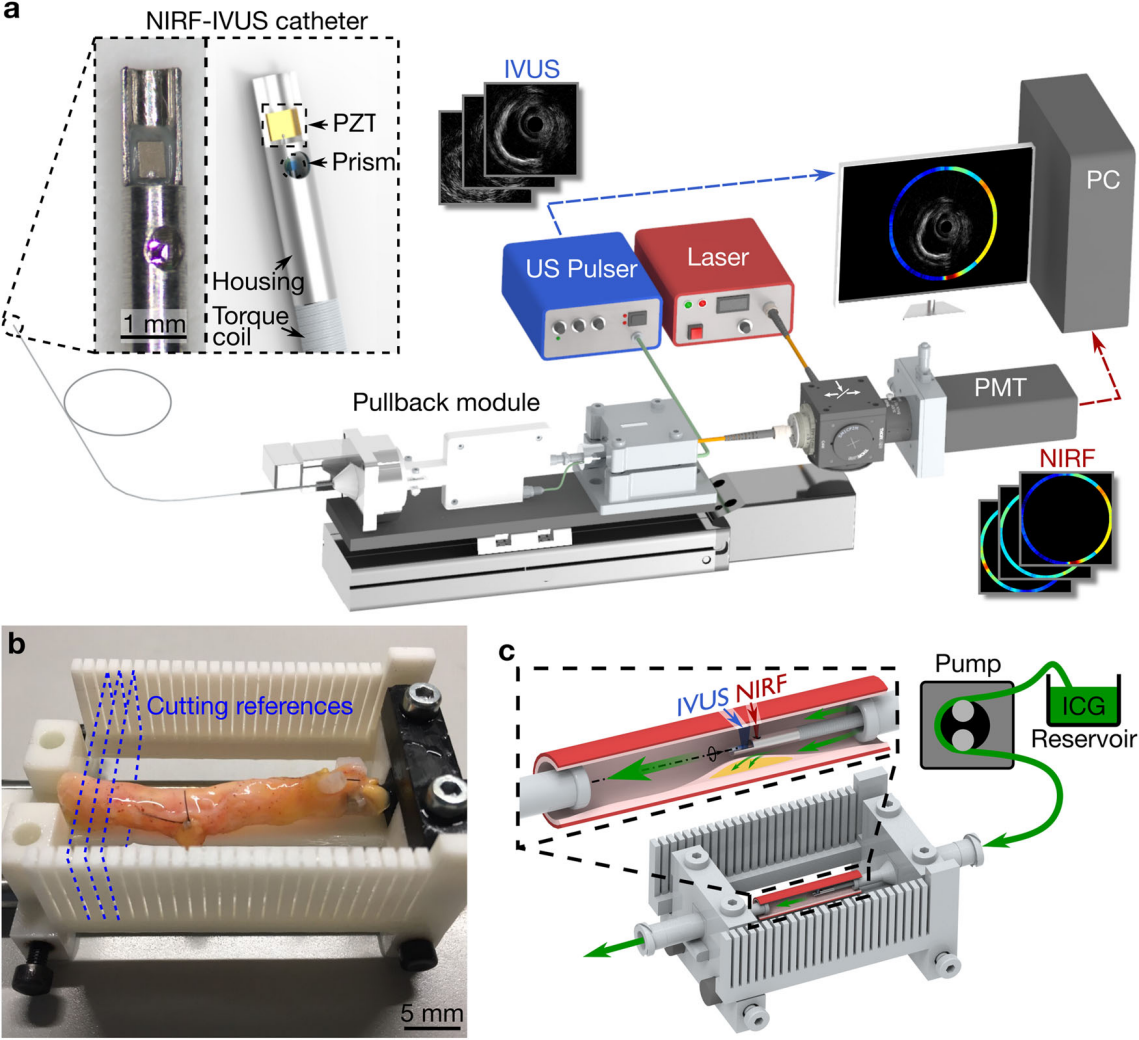
Intravascular imaging and ex vivo perfusion setup. **a** 3.7 F NIRF-IVUS imaging catheter, pullback module and acquisition system; Adapted from^5,28^. **b** Excised human coronary artery in 3D-printed holder that facilitates sectioning to allow for co-registration of imaging data and histological images. **c** Ex vivo ICG perfusion of human coronary arteries fixed in a holder for intravascular NIRF-IVUS imaging. ICG indocyanine green, IVUS intravascular ultrasound, NIRF near-infrared fluorescence, PMT photomultiplier tube, PZT piezoelectric element, US ultrasound; Some computer assisted design models are courtesy of Thorlabs (www.thorlabs.com).

We developed a hybrid NIRF-IVUS imaging system based on a setup previously reported by our group^5,7,28^. The system comprised a 3.7 F NIRF-IVUS imaging catheter and a complementary back-end system, which consists of a control and readout system and a helical pullback module (Fig. 5a).

Our NIRF-IVUS catheter features a micro prism attached to a 200/220 μm multimode fiber and a piezoelectric element (size: 0.6 × 0.5 mm; center frequency: 40 MHz; axial resolution: 40 μm; lateral resolution: 120 μm; Blatek, Boalsburg, Pennsylvania, USA) for NIRF-IVUS signal excitation and detection. The catheter is inserted into a size 3.7 F (Outer diameter: 1.24 mm) sheath which was custom-made out of tubing (Material: Low-density polyethene, ZEUS, Orangeburg, USA) connected to a 7 F introducer sheath (RT-R70D10PQ, Terumo Europe) functioning as a flush port.

A custom-made LabVIEW (National Instruments Corp., Austin, Texas, USA) software was implemented for system control and data acquisition, while all data were analyzed in MATLAB (Mathworks, Natick, Massachusetts, USA).

### Indocyanine Green (ICG) perfusion and ex vivo intravascular imaging

Written informed consent was provided by family members of patients deceased at the *Deutsches Herzzentrum Muenchen* or *1. Medizinische Klinik, Klinikums rechts der Isar, Technical University of Munich (TUM)*. This study was approved by the ethics committee of the Technical University of Munich (reference number: 291/18 S). Within 48 hours of death, coronary arteries (*n* = 15) were excised from the donor’s heart (total *n* = 11) and flushed with cold PBS. The clinical characteristics like sex, age, cause of death, and medical history of the donors are summarized in Table S1. Coronary artery segments were prepared by trimming off excessive adjacent fat and ligating side branches. An exclusion criterion for the imaging study was the presence of a high degree of luminal stenosis, to lower the risk of tissue damage potentially caused by the insertion of the imaging catheter. The average length of a coronary artery segment was 33 mm (Range between 9-50 mm) after preparations were completed.

Next, arteries were mounted in a custom-made holder (as in ref. ^29^, Fig. 5b), which enabled precise angular and axial co-registration between NIRF-IVUS and histological images (see Supplemental information 1 and Fig. S6). A peristaltic pump (Ecoline, ISMATEC, Cole-Parmer, Wertheim, Germany) perfused the coronary arteries for 5 minutes (similar to ICG’s blood half-life in vivo^16^) with 500 ml of ICG (VERDYE, Diagnostic Green, Aschheim-Dornach, Germany) which was dissolved in water to a final concentration of 20 μM (Fig. 5c). After ICG perfusion, coronary arteries were flushed with 500 ml of cold PBS for 5 minutes to remove unbound ICG, mounted into a PBS bath, and imaged with intravascular NIRF-IVUS while perfusing PBS through the arterial lumen. All NIRF-IVUS data were acquired with 142 rpm rotation and 0.5 mm/s pullback speeds for various distances ranging between 15-50 mm (i.e. 60-200 cross-sectional NIRF-IVUS frames per artery). After NIRF-IVUS imaging was completed, arteries were divided into two groups for further processing.

### Fluorescence microscopy and histology

The first sub-group of coronary arteries (*n* = 4) was sectioned transversely at 2 mm intervals, embedded in optimal cutting temperature compound (Tissue-Tek, Sakura Finetek, Staufen, Germany) and flash-frozen using dry ice immediately after NIRF-IVUS imaging. Representative sections were cut at 10 μm thickness and a fluorescence microscope (BX41, Olympus, Tokyo Japan; Camera: C11440-36U, Hamamatsu, Japan) was used to detect ICG (ICG channel, Excitation/Emission 730/776 nm) and autofluorescence (FITC channel, Excitation/Emission 495/520 nm) within arterial tissue. Cryosections were stained with MP and Oil Red O (ORO) staining. Immunostaining was performed using antibodies against CD68 (Dako, M0718), alpha-smooth muscle actin (αSMA, abcam, ab5694) and CD31 (Dako, M0823). The second sub-group (*n* = 11) was kept in the artery holder and fixed in formalin for 24-48 before additional imaging with a commercially available IVOCT system (Abbot, Dragonfly OPTIS, Plymouth, USA) to support co-registration of imaging findings to histology according to lumen contour. After IVOCT imaging, arteries were sectioned transversely at 2 mm intervals and resulting sub-segments were embedded in paraffin. Sections were cut to 10 μm thickness and stained with hematoxylin and eosin (H&E), MP, vK and marked with CD68, CD31 and αSMA antibodies.

### NIRF-IVUS imaging analysis

NIRF-IVUS data were post-processed in MATLAB (Version R2023a); post-processing included normalization, correction of NIRF signal due to changes in distance between the sensor and artery wall (see Supplemental information 2 and Fig. S7), conversion of NIRF intensity to ICG-concentration, and creation of hybrid NIRF-IVUS cross-sectional images.

Histological sections of arteries from both sub-groups were divided into 30° sectors (*n* = 295 from the first and *n* = 792 from the second sub-group) and each sector was classified as (i) early-stage intimal lesions corresponding to intimal xanthoma or PIT without extracellular lipid pool, (ii) PIT with extracellular lipid pool, (iii) fibroatheroma, (iv) fibrous/fibrocalcific plaque or (v) luminal calcification^24^. Lesions were classified by a cardiologist blinded to the NIRF-IVUS and FM data.

The paraffin-embedded sub-group of arteries was used to investigate the correlation of ICG-derived NIRF signals to signs of inflammation. The analysis was performed in MATLAB: Image scans of co-registered histology sections stained for macrophages using CD68 were binarized using a pre-defined pixel-threshold range for the individual channels in the HSV color space (H:0–1; S: 0.164-1; V:0-0.826). Using representative histology sections, the threshold range was determined prior to the analysis marking CD68-positive pixels as 1 and all remaining as 0. This pixel threshold range was kept constant for all CD68 stained sections included in the analysis. Next, binarized images were transformed from cartesian to polar coordinates to (i) define a ROI measured at a tissue depth of 500 µm from the artery lumen and (ii) divide NIRF and histology data in co-registered 30° sectors (S_1_-S_12_, *n* = 792) for further analysis. The ROI depth was chosen to focus the analysis on intima macrophages and exclude CD68 staining artifacts and tissue ink located in fat tissue adjacent to the adventitia. Finally, the density of CD68-positive pixels ((Number of pixels = 1)/(Number of pixels = 0)) in the ROI for each sector was calculated and correlated to the average ICG-concentration detected in the same sector by NIRF-IVUS which resulted in the Pearson correlation coefficient (r).

### Statistical analysis

Categorical data were expressed as counts or percentages. Continuous data were checked for normality of distribution using Wilk-Shapiro test and expressed as means with standard deviation in case of normal distribution and median with interquartile range in case of non-parametric distribution. Analysis was carried out using IBMS SPSS Version 26.0 software (IBM Corp., Armonk, New York, USA). To account for the clustered nature of the data, a linear mixed model was used to perform correlations to assess the relationship between average ICG-concentration detected by NIRF-IVUS per sector and the corresponding pathological classifications. The model contained a fixed-effects term for the variable of interest (stage of atherosclerosis) and a random intercept to estimate the random-effects term for the cross-sectional analysis of histology sections from the same artery. The correlation between detected ICG-concentration and density of CD68-positive pixels was evaluated by a leave-one-out cross-validation approach, whereby a full artery segment was removed for each fold^29^. The consistency of the analysis across folds was evaluated by reporting the averaged variance between folds. P < 0.05 was considered statistically significant.

## Supplementary information

Supplementary Information

## Data Availability

The datasets used and/or analyzed during the current study are available from the corresponding author on reasonable request.

## Code availability

The underlying code for this study is not publicly available but may be made available to qualified researchers on reasonable request from the corresponding author.
