# Supplementary material for: Intravascular ICG-enhanced NIRF-IVUS imaging to assess progressive atherosclerotic lesions in excised human coronary arteries
Source: NPJ Cardiovasc Health. 2024 Aug 30;1:14. doi: 10.1038/s44325-024-00016-8 (PMC11378621; doi:10.1038/s44325-024-00016-8)
Supplement: Supplementary file 1 — Supplementary Information [file 44325_2024_16_MOESM1_ESM.pdf]

Supplemental Information

1. | Co-registration between NIRF-IVUS imaging data and histology

The analyses done in this work required precise co-registration between intravascular NIRF-IVUS data and histological images. Thus, angular markers were installed, which were visible in the imaging data and histology to enable accurate angular co-registration. Specifically, the artery holder featured a structure that creates a reference signal at the 180° position (Blue box) in all hybrid NIRF-IVUS frames (Figure S6a). Furthermore, additional IVOCT imaging pullbacks were acquired to provide detailed information about the coronary lumen to support co-registration with histology (Figure S6b). Due to limited penetration depth of IVOCT, the angular maker of the artery holder was not visible in the IVOCT images. Thus, a coronary guidewire was inserted into artery holder to create a reference signal at the 180° position (Blue box) at the end of the IVOCT imaging pullbacks (Figure S6b). The pullback position provided information for the axial co-registration for both, NIRF-IVUS and IVOCT imaging data. After completion of intravascular imaging, arteries were marked at the 0°/360° position with tissue ink to provide an indicator for angular orientation (Figure S6c). Furthermore, cutting references were implemented into the holder (Figure 5b) to enable transverse sections at 2-3 mm intervals affording several subsegments for axial co-registration. The combined information of axial and angular markers in both, the imaging frames and histology section, allowed precise co-registration of 1087 sectors affording an angular resolution of 30°. This accuracy also allowed us to quantify severity of tissue inflammation based on an automated post-processing algorithm, which avoided potential bias and facilitated the use of a fully quantitative scale in contrast to most semi-quantitative assessments applied in conventional

histopathological image analysis (described in the Methods). Figure S5 provides an overview of IVOCT images recorded in coronary segments of the second sub-group which were co-registered to NIRF-IVUS images presented in Figures 2 and 4.

2. | Normalization, distance correction and sensitivity limit of intravascular NIRF signals.

NIRF intensities of all datasets were normalized to each other to account for small differences in ICG concentration used for perfusing the arteries and potential changes in NIRF sensitivity of the system during the time period of data collection due to e.g. alignment changes in the optical detection path or contamination of the NIRF sensor located in the catheter. This normalization ensured that datasets were comparable to each other. For this purpose, a sample of each ICG perfusate was filled into a glass-capillary prior to every measurement and was imaged with the NIRF-IVUS system to record NIRF reference values. After the NIRF-IVUS image acquisition for all coronary arteries was completed, reference values were used to normalize the individual datasets to highest NIRF intensity value recorded during all reference measurements. NIRF background caused by fiber autofluorescence was quantified by calculating average intensities recorded for each tissue pullback when the NIRF-IVUS catheter was located outside the perfused coronary segments in the water bath. NIRF background was regarded as noise to calculate tissue signal-to-noise ratios.

Furthermore, intravascular NIRF data needs to be corrected for signal variation due to changes in distance between the sensor and the artery wall throughout the imaging pullback. This correction removes the influence of the distance on the detected NIRF signals and allows conversion of NIRF intensity to ICG concentration values. For this

purpose, a distance-dependent correction model, based on phantom measurements of an ICG-Lipid-Agar patch, was constructed. In contrast to NIRF measurements of ICG dissolved in a transparent solution like saline, NIRF measurements of ICG dissolved in the Lipid-Agar phantom resembled the diffusive optical properties expected in human tissue more closely[1-2]. The patch was created by heating 20% Intralipid (I141, Sigma-Aldrich) to 90°C and dissolving 3% of Agar (A0950, Agar, Noble). The mixture was then cooled down at room-temperature while continuously mixing. At around 45°C, 10 µL of ICG (dissolved in DMSO) was added to a final concentration of 10.7 µM. The liquid mixture was then poured at a temperature of 40°C between two microscope cover slips to solidify and create the final patch. The patch was then placed in a tilted position into a 3D-printed phantom to record NIRF-IVUS measurements through saline at variable distances between the patch and the NIRF-IVUS catheter (Figure S7a). Figure S7b shows the NIRF map of the ICG-Lipid-Agar patch acquired during the imaging pullback and figure S7c displays a representative IVUS frame of the patch in polar coordinates indicating the distance, d, between the catheter sheath and the surface of the patch. The measurements were repeated 4 times and averaged NIRF intensities (Highest 5 values averaged per frame) were then plotted versus the distance from the sheath to the patch (quantified by IVUS) to fit a two-term exponential function[3-5] (Figure S7d, black) resulting in the distance-dependent correction function α(d), (Eq. 1):

$$\alpha(d)= 281.7*e^{(-0.0027*d)}+593.8*e^{(-0.000355*d)}. \quad (1)$$

This function was then used to correct NIRF signals acquired from coronary artery tissue for distance between sheath and the coronary artery wall (Quantified by IVUS) according to (Eq. 2):

$$I_{Cor} = \frac{I_{Raw}}{\alpha(d)} \quad (2)$$

where I_{Cor} is the distance corrected NIRF intensity and I_{Raw} is the uncorrected NIRF intensity measured during NIRF-IVUS imaging pullbacks of the coronary arteries. Finally, I_{Cor} values were converted into ICG concentration by referring to the known ICG concentration in the Lipid-Agar patch. In addition, we repeated phantom measurements through whole pig blood to estimate the NIRF sensitivity of the system. Figure S7d (Red) shows NIRF SNR calculated from fluorescence intensities recorded through blood. We confirmed that ICG in the phantom was detectable by the NIRF-IVUS catheter (SNR_{2mm} = 1.8) through 2 mm of blood. We consider these results as the minimum sensitivity limit for NIRF imaging in tissue since NIRF signal attenuation is expected to be lower in tissue than in blood[6].

Supplemental table

	Value
**Number, n**	11
**Sex**	
male, n	5
female, n	6
**Age in years, mean ± SD**	76.7 ± 7.7
**Cause of death, n (%)**	
Cardiovascular	3 (27)
STEMI	2 (18)
Heart failure	1 (9)
Non-cardiovascular	7 (63)
Pulmonary cause	4 (36)
Sepsis	2 (18)
Haemorrhage	1 (9)
Unknown	1 (9)
**Medical history, n (%)**	
Hypertension	6 (54)
Current or former smoker	2 (18)
Diabetes Mellitus	2 (18)
Prior MI	2 (18)
Prior CABG	1 (9)

Table S1: **Clinical characteristics of coronary artery specimen donors.** STEMI ST-Elevation myocardial infraction; MI Myocardial infraction; CABG Coronary artery bypass graft.

Supplemental references

1. Pogue, B.W. & Patterson, M.S. Review of tissue simulating phantoms for optical spectroscopy, imaging and dosimetry. *J Biomed Opt*. **11**, 041102 (2006).
2. Cubeddu, R., Pifferi, A., Taroni, P., Torricelli, A. & Valentini G. A solid tissue phantom for photon migration studies. *Phys Med Biol*. **42,** 1971-1979 (1997).
3. Kellnberger, S., Wissmeyer, G., Albaghdadi, M., Piao, Z., Li, W., et al. Intravascular molecular-structural imaging with a miniaturized integrated near-infrared fluorescence and ultrasound catheter. *J Biophotonics*. **14**, e202100048 (2021).
4. Rauschendorfer, P., Wissmeyer, G., Jaffer, F.A., Gorpas, D & Ntziachristos, V. Accounting for blood attenuation in intravascular near-infrared fluorescence-ultrasound imaging using a fluorophore-coated guidewire. *J Biomed Opt*. **28**, 046001(2023).
5. Bozhko, D., Karlas, A., Gorpas, D. & Ntziachristos, V. Optoacoustic sensing of hematocrit to improve the accuracy of hybrid fluorescence-ultrasound intravascular imaging. *J Biophotonics*. **11**, 201700255 (2018).
6. Jaffer, F.A., Vinegoni, C., John, M.C., Aikawa, E., Gold, H.K. Real-Time Catheter Molecular Sensing of Inflammation in Proteolytically Active Atherosclerosis. *Circulation*. **18**, 1802-1809 (2008).

Supplemental figures

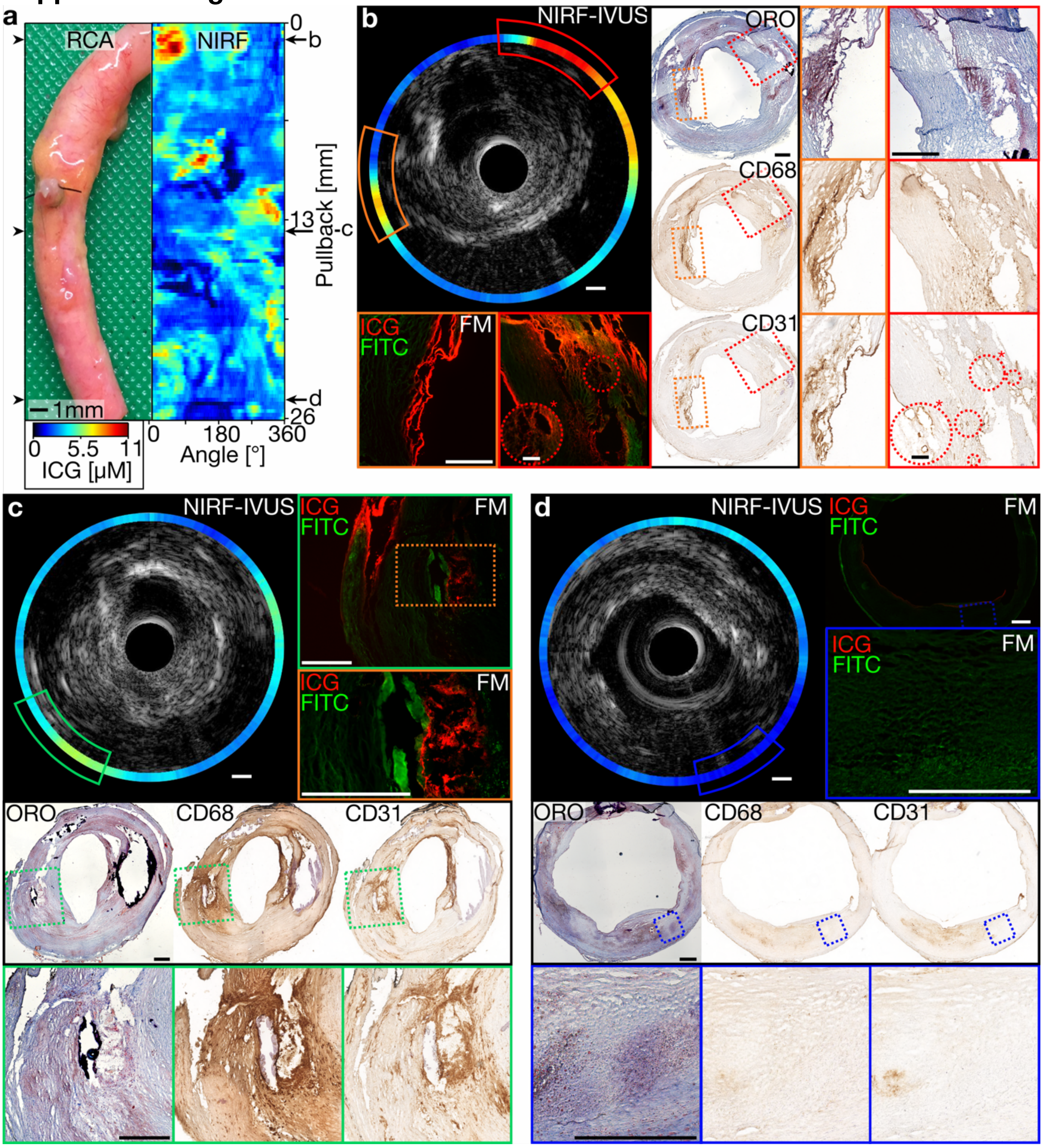

Figure S1: **NIRF-IVUS imaging of an ICG-perfused coronary artery detected increased ICG concentrations spatially related to plaque areas with accumulated lipids, infiltrated macrophages and neovascularization. (a)** Picture and NIRF map of an ICG-perfused RCA with representative pullback locations co-registered to tissue cryosections for histological assessment and confirmation of ICG accumulation by FM (b-d); **(b)** Cross-sectional NIRF-IVUS image showcasing high

ICG concentrations (NIRF-IVUS - red and orange box) co-localized to plaque regions with accumulated lipids (ORO – red and orange box), infiltrated macrophages (CD68 – red and orange box) and the presence of neovascularization (CD31 and FM – Co-registered areas of neovascularization shown as circles in red box; Scale bar zoom in*: 100 μm); **(c)** Cross-sectional NIRF-IVUS image indicating increased ICG concentrations (NIRF-IVUS - green box) in a coronary plaque region featuring a lipid-rich (ORO - green box) necrotic core with infiltrated macrophages (CD68 – green box); **(d)** Cross-sectional NIRF-IVUS image showing low ICG concentration (NIRF-IVUS - blue box) in a tissue region of PIT without accumulated lipids (ORO - blue box), infiltrated macrophages (CD68 – blue box) and neovascularization (CD31 – blue box). In all panels: FITC Fluorescein channel for autofluorescence; FM Fluorescence Microscopy; ICG Indocyanine Green; IVUS Intravascular Ultrasound; NIRF Near-infrared Fluorescence; ORO Oil Red O; PIT Pathological intimal thickening. Scale bars unless otherwise specified: 500 μm.

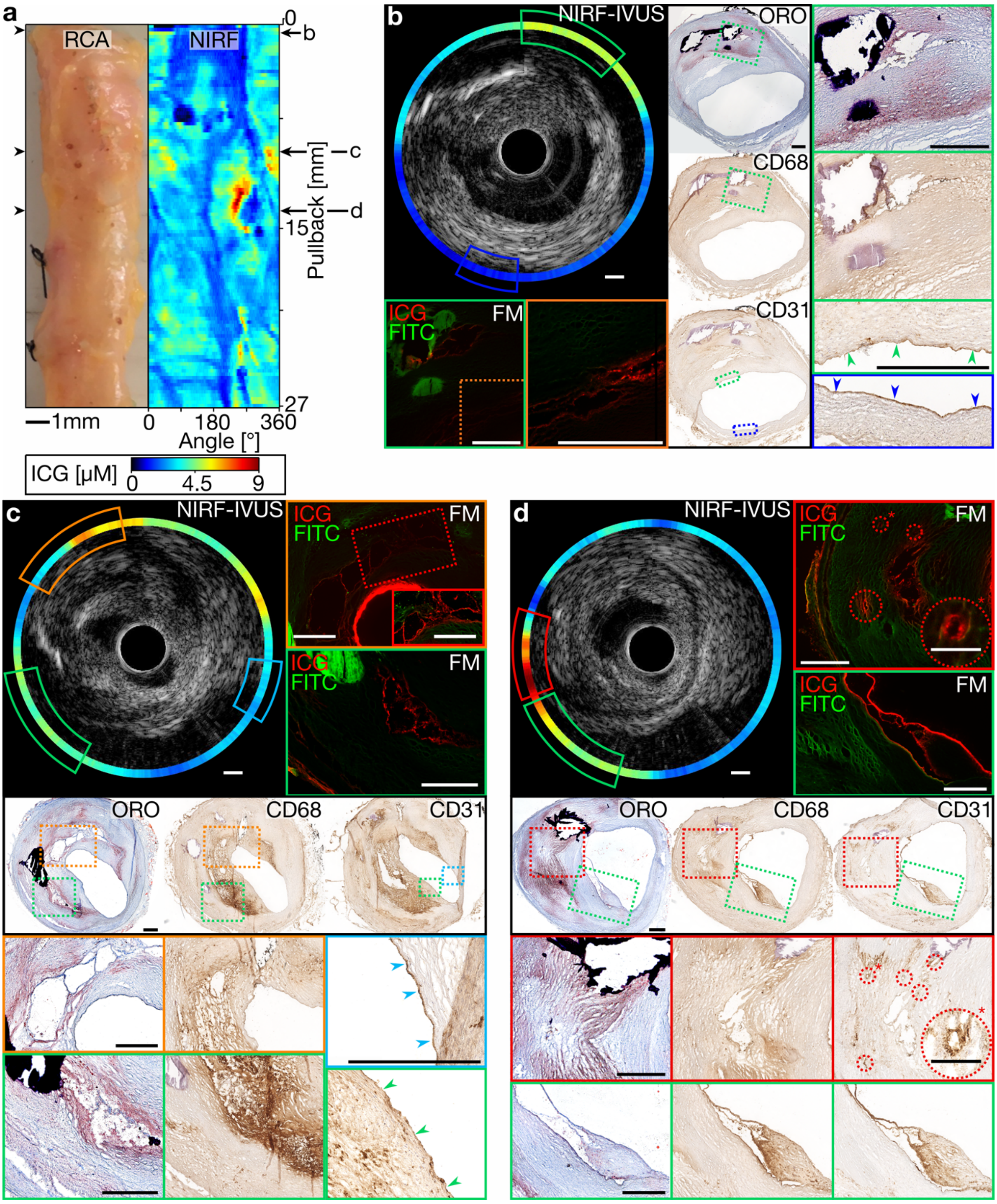

Figure S2: **NIRF-IVUS imaging of an ICG-perfused coronary artery detects high ICG concentrations spatially related to plaque areas with accumulated lipids, infiltrated macrophages, endothelial disruption, and neovascularization. (a)** Picture and NIRF map of an ICG-perfused RCA with representative pullback locations co-registered with tissue cryosections for histological assessment and confirmation

of ICG accumulation by FM (b-d); **(b)** Cross-sectional NIRF-IVUS image showcasing mildly increased ICG concentrations (NIRF-IVUS - green box) co-localized to plaque regions with accumulated lipids (ORO - green box), no significant macrophage infiltration (CD68 - blue box) and absent endothelial cells (CD31 – green box; green arrows indicating disrupted endothelium). Low ICG concentrations (NIRF-IVUS - blue box) found in a tissue region of early-stage intimal lesion with present endothelial cells (CD31 – blue box; blue arrows indicating undisrupted endothelium); **(c)** Cross-sectional NIRF-IVUS image showing increased ICG concentrations (NIRF-IVUS - orange and green box) co-localized to plaque regions with accumulated lipids (ORO - green box), infiltrated macrophages (CD68 – orange and green box) and absent endothelial cells (CD31 – green box; green arrows indicating disrupted endothelium). Low ICG concentration (NIRF-IVUS – light blue box) found in a tissue region of early-stage intimal lesion with present endothelial cells (CD31 - light blue box; light blue arrows indicating undisrupted endothelium); **(d)** Cross-sectional NIRF-IVUS image showing high ICG concentrations (NIRF-IVUS - red and green box) co-localized to plaque regions with accumulated lipids (ORO - red box), infiltrated macrophages (CD68 – green box) and neovascularization (CD31 and FM – Co-registered areas of neovascularization shown as circles in red box; Scale bar zoom in*: 100 µm). In all panels: FITC Fluorescein channel for autofluorescence; FM Fluorescence Microscopy; ICG Indocyanine Green; IVUS Intravascular Ultrasound; ORO Oil Red O; NIRF Near-infrared Fluorescence; Scale bars unless otherwise specified: 500 µm.

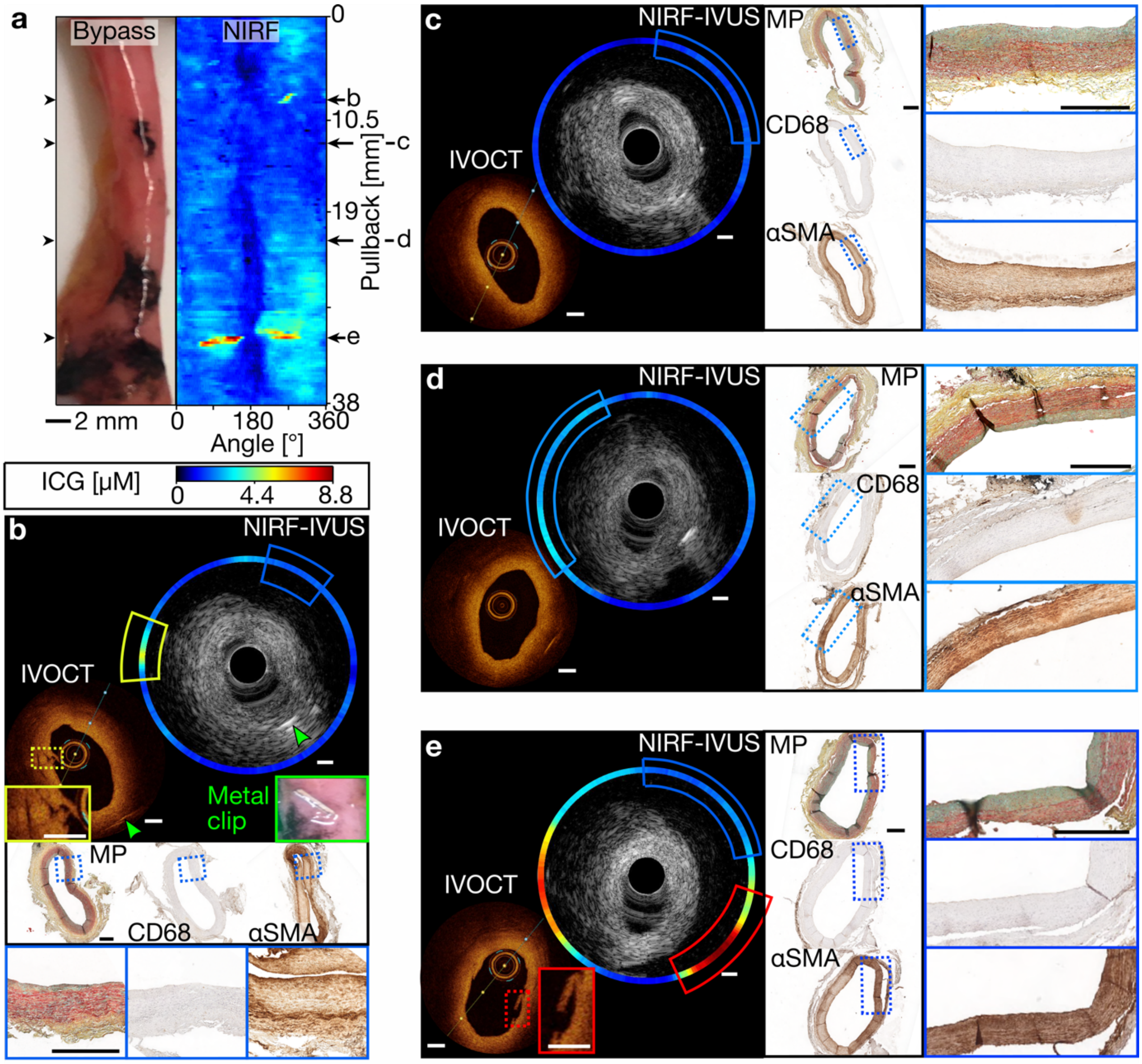

Figure S3: **NIRF-IVUS imaging of an ICG-perfused coronary bypass detected low ICG concentrations in early-stage intimal lesions. (a)** Picture and NIRF map of an ICG-perfused bypass with representative pullback locations co-registered to tissue sections for histological assessment (b-e); **(b)** Cross-sectional NIRF-IVUS image showing low ICG concentrations (NIRF-IVUS - blue box) co-localized to a tissue region of mildly thickened intima (MP – blue box), the presence of smooth-muscle cells (αSMA – blue box) and without the presence of macrophages (CD68– blue box). Increased ICG concentration (NIRF-IVUS – yellow box) detected in a tissue area showing signs of tissue disruption visible in co-registered (Metal clip used as

additional angular marker – green arrow) IVOCT image (IVOCT - yellow box); **(c-d)** Cross-sectional NIRF-IVUS image showing low ICG concentrations (NIRF-IVUS - blue box) in a tissue region of early-stage intimal lesions with mildly thickened intima (MP – Blue box), the presence of smooth-muscle cells (αSMA – blue box) and without the presence of macrophages (CD68– blue box). Co-registered IVOCT images show no signs of pathological changes. **(e)** Cross-sectional NIRF-IVUS image showing low ICG concentrations (NIRF-IVUS - blue box) co-localized to a tissue region of mildly thickened intima (MP – blue box), the presence of smooth-muscle cells (αSMA – blue box) and without the presence of macrophages (CD68 – blue box). Increased ICG concentration (NIRF-IVUS – red box) detected in a tissue area showing signs of tissue disruption visible in co-registered (IVOCT image (IVOCT - red box); In all panels: ICG Indocyanine Green; IVOCT Intravascular Optical Coherence Tomography; IVUS Intravascular Ultrasound; MP Movat's Pentachrome; NIRF Near-infrared Fluorescence; αSMA alpha-smooth muscle actin; Scale bars: 500 μm.

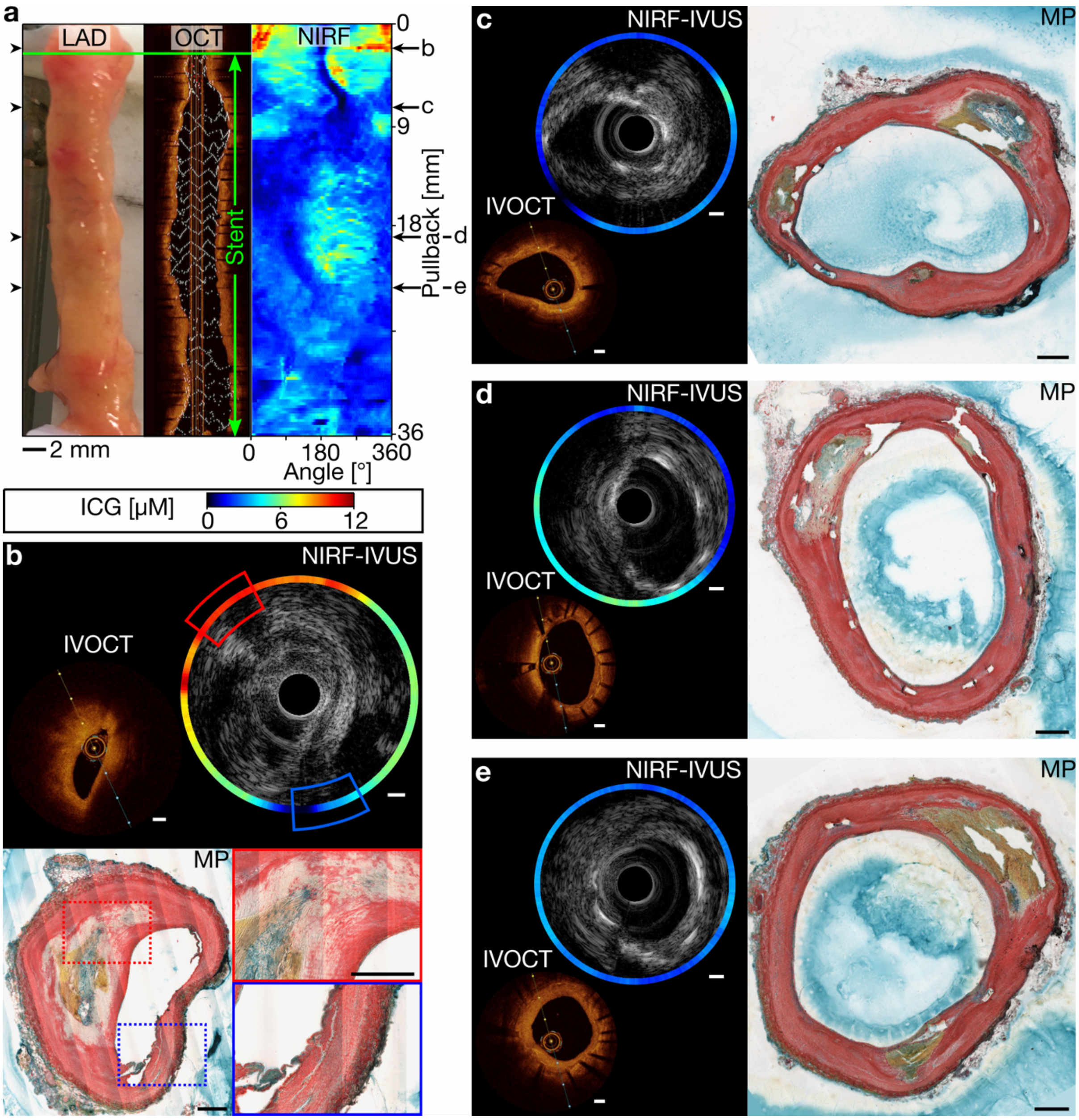

Figure S4: **NIRF-IVUS imaging of an ICG-perfused coronary artery detected high ICG concentration in plaque area without stent coverage and low ICG concentrations in stented tissue regions. (a)** Picture, IVOCT and NIRF map of an ICG-perfused LAD indicating stented tissue region between the pullback positions 3mm and 36mm and representative pullback locations co-registered with consecutive MMA-embedded tissue sections stained for histological assessment (b-e); **(b)** Cross-sectional NIRF-IVUS image showcasing high ICG concentration (NIRF-IVUS - red box) co-localized to a plaque area identified as late-stage fibroatheroma

(MP – red box) in contrast to low ICG concentrations (NIRF-IVUS - blue box) detected in tissue regions of early-stage lintimal esions (MP – blue box) without stent coverage; **(c-e)** Cross-sectional NIRF-IVUS images showing low ICG concentrations (NIRF-IVUS) in stented tissue regions with underlying fibroatheroma fully covered by undisrupted neo-endothelium (MP); In all panels: ICG Indocyanine Green; IVOCT Intravascular OCT; IVUS Intravascular Ultrasound; MP Movat's Pentachrome; NIRF Near-infrared Fluorescence; Scale bars: 500 μm.

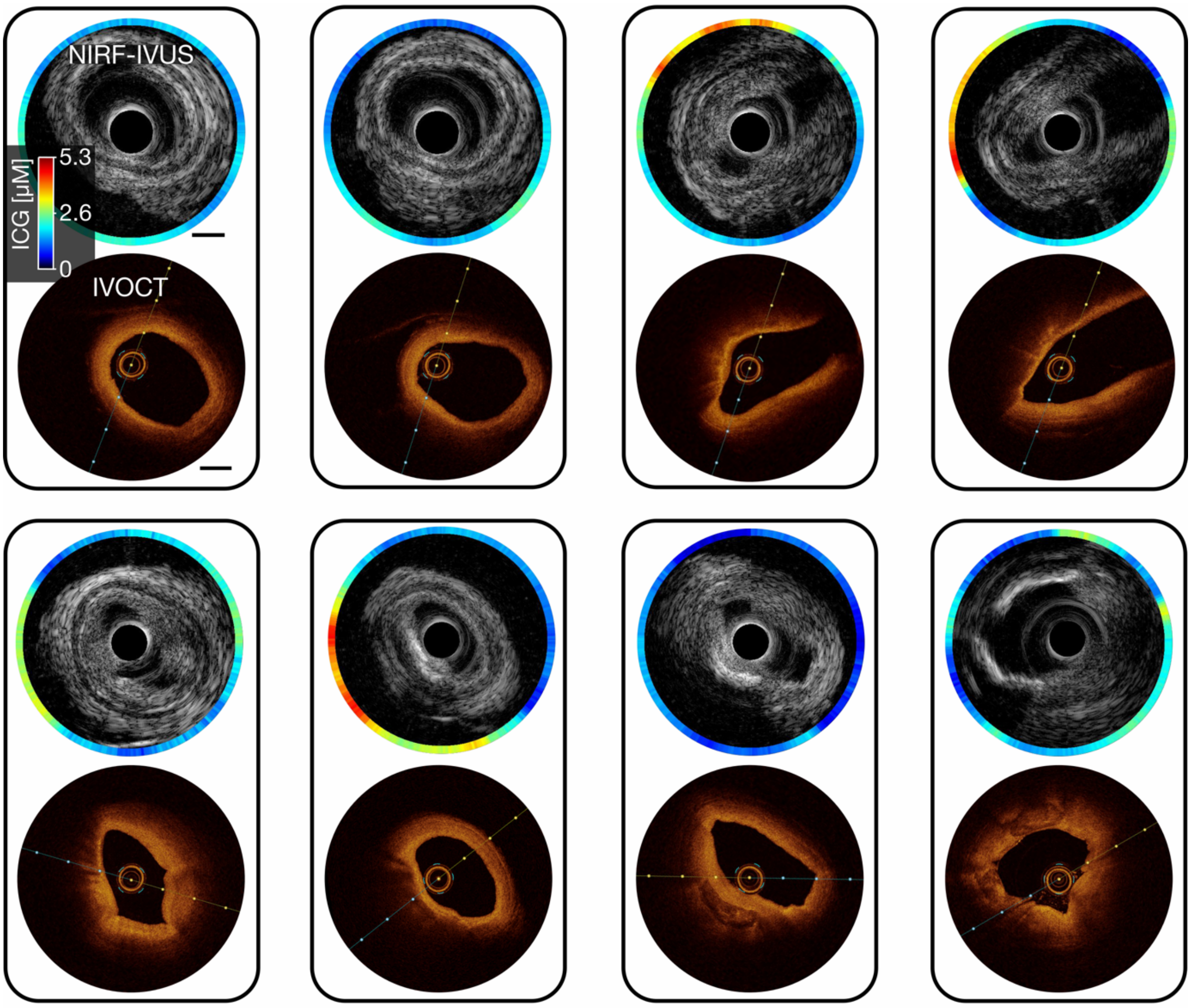

Figure S5: **IVOCT co-registered to NIRF-IVUS images;** IVOCT Intravascular optical coherence tomography; IVUS Intravascular Ultrasound; NIRF Near-infrared Fluorescence; Scale bars: 1 mm.

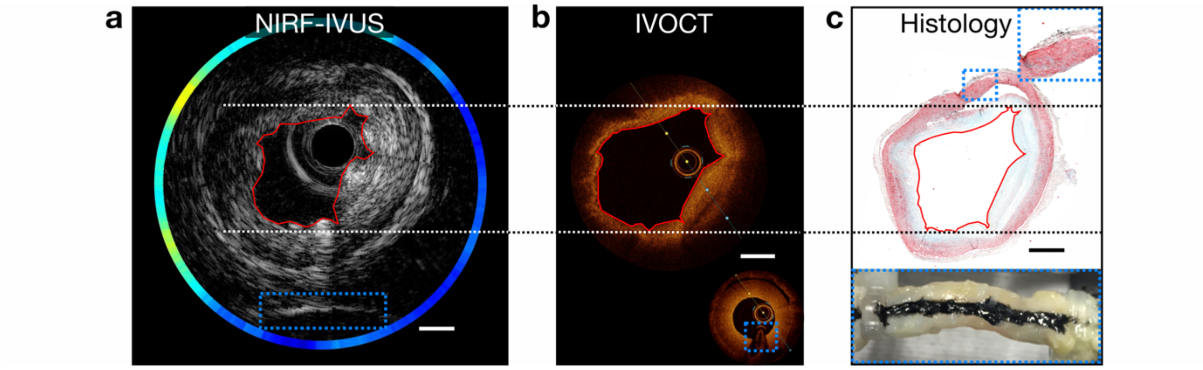

Figure S6: **Angular co-registration between intravascular NIRF-IVUS imaging data and histological image.** **(a)** Representative NIRF-IVUS frame of coronary tissue showcasing IVUS reference signal (Blue box) as an angular marker at the 180° location and; **(b)** Co-registered IVOCT frame of coronary tissue showcasing IVOCT reference signal of an inserted guidewire (Blue box) as an angular marker at the 180° location; **(c)** Co-registered histology section stained with MP and picture of an artery segment indicating black tissue ink as an angular marker at the 0°/360° location (Blue boxes); In all panels: Lumen was outlined in red; IVOCT Intravascular optical coherence tomography; IVUS Intravascular Ultrasound; MP Movat's pentachrome; NIRF Near-infrared Fluorescence; Scale bars: 1 mm.

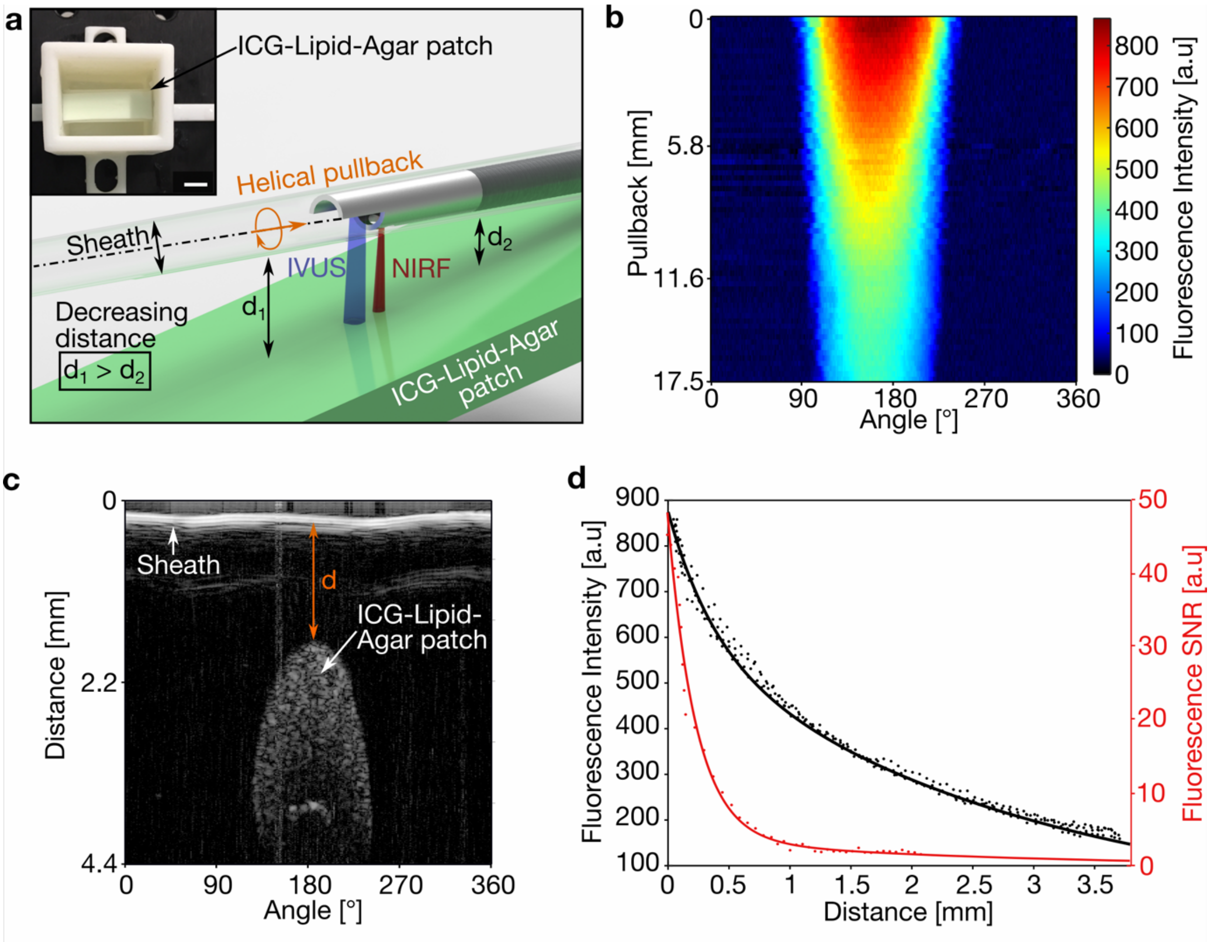

Figure S7: **Development of a distance-dependent correction model for intravascular NIRF signals using an ICG-lipid-agar phantom. (a)** Picture and illustration of a phantom with an ICG-Lipid-Agar patch measured at variable distances (d) to the NIRF-IVUS catheter; Scale bar: 5 mm; **(b)** NIRF map of ICG-Lipid-Agar patch for one imaging pullback through saline; **(c)** Exemplary IVUS frame of the phantom measurements in polar coordinates indicating the distance, d, between the catheter sheath and the ICG-Lipid-Agar patch; **(d)** Results of the phantom measurements; Fluorescence intensity recorded through saline (black) and SNR calculated from measurements recorded through blood (red) at variable distances with corresponding exponential model fits; In all panels: ICG Indocyanine Green; IVUS Intravascular Ultrasound; NIRF Near-infrared Fluorescence.

nature portfolio

Corresponding author(s): Vasilis Ntziachristos

Last updated by author(s): Philipp Rauschendorfer on 25.04.24

Reporting Summary

Nature Portfolio wishes to improve the reproducibility of the work that we publish. This form provides structure for consistency and transparency in reporting. For further information on Nature Portfolio policies, see our Editorial Policies and the Editorial Policy Checklist.

Please do not complete any field with "not applicable" or n/a. Refer to the help text for what text to use if an item is not relevant to your study.
For final submission: please carefully check your responses for accuracy; you will not be able to make changes later.

Statistics

For all statistical analyses, confirm that the following items are present in the figure legend, table legend, main text, or Methods section.

n/a	Confirmed	
☐	☒	The exact sample size (*n*) for each experimental group/condition, given as a discrete number and unit of measurement
☐	☒	A statement on whether measurements were taken from distinct samples or whether the same sample was measured repeatedly
☐	☒	The statistical test(s) used AND whether they are one- or two-sided<br>*Only common tests should be described solely by name; describe more complex techniques in the Methods section.*
☐	☒	A description of all covariates tested
☐	☒	A description of any assumptions or corrections, such as tests of normality and adjustment for multiple comparisons
☐	☒	A full description of the statistical parameters including central tendency (e.g. means) or other basic estimates (e.g. regression coefficient) AND variation (e.g. standard deviation) or associated estimates of uncertainty (e.g. confidence intervals)
☐	☒	For null hypothesis testing, the test statistic (e.g. *F*, *t*, *r*) with confidence intervals, effect sizes, degrees of freedom and *P* value noted<br>*Give P values as exact values whenever suitable.*
☒	☐	For Bayesian analysis, information on the choice of priors and Markov chain Monte Carlo settings
☒	☐	For hierarchical and complex designs, identification of the appropriate level for tests and full reporting of outcomes
☐	☒	Estimates of effect sizes (e.g. Cohen's *d*, Pearson's *r*), indicating how they were calculated

Our web collection on statistics for biologists contains articles on many of the points above.

Software and code

Policy information about availability of computer code

Data collection	The underlying code for this study is not publicly available but may be made available to qualified researchers on reasonable request from the corresponding author.
Data analysis	The underlying code for this study is not publicly available but may be made available to qualified researchers on reasonable request from the corresponding author.

For manuscripts utilizing custom algorithms or software that are central to the research but not yet described in published literature, software must be made available to editors and reviewers. We strongly encourage code deposition in a community repository (e.g. GitHub). See the Nature Portfolio guidelines for submitting code & software for further information.

Data

Policy information about availability of data

All manuscripts must include a data availability statement. This statement should provide the following information, where applicable:

- Accession codes, unique identifiers, or web links for publicly available datasets
- A description of any restrictions on data availability
- For clinical datasets or third party data, please ensure that the statement adheres to our policy

The datasets used and/or analyzed during the current study are available from the corresponding author upon reasonable request.

Research involving human participants, their data, or biological material

Policy information about studies with human participants or human data. See also policy information about sex, gender (identity/presentation), and sexual orientation and race, ethnicity and racism.

Reporting on sex and gender	We are reporting on six female and five male patients.
Reporting on race, ethnicity, or other socially relevant groupings	N/A
Population characteristics	Population characteristics are summarized in the supplementary information, Table S1.
Recruitment	Written informed consent was provided by family members of patients deceased at the Deutsches Herzzentrum Muenchen or 1. Medizinische Klinik, Klinikums rechts der Isar, Technical University of Munich (TUM).
Ethics oversight	This study was approved by the ethics committee of the Technical University of Munich (reference number: 291/18 S)

Note that full information on the approval of the study protocol must also be provided in the manuscript.

Field-specific reporting

Please select the one below that is the best fit for your research. If you are not sure, read the appropriate sections before making your selection.

☒ Life sciences ☐ Behavioural & social sciences ☐ Ecological, evolutionary & environmental sciences

For a reference copy of the document with all sections, see nature.com/documents/nr-reporting-summary-flat.pdf

Life sciences study design

All studies must disclose on these points even when the disclosure is negative.

Sample size	15 coronary artery specimens excised from 11 donors' hearts.
Data exclusions	An exclusion criterion for the imaging study was the presence of a high degree of luminal stenosis, to lower the risk of tissue damage potentially caused by the insertion of the imaging catheter
Replication	Image scans of coronary artery specimens were not repeated to avoid photobleaching of ICG
Randomization	Randomization was not applied.
Blinding	The researcher responsible for lesion classification was blinded to NIRF-IVUS and fluorescence microscopy data.

Behavioural & social sciences study design

All studies must disclose on these points even when the disclosure is negative.

Study description	
Research sample	
Sampling strategy	
Data collection	
Timing	
Data exclusions	
Non-participation	
Randomization	

Ecological, evolutionary & environmental sciences study design

All studies must disclose on these points even when the disclosure is negative.

Study description	
Research sample	
Sampling strategy	
Data collection	
Timing and spatial scale	
Data exclusions	
Reproducibility	
Randomization	
Blinding	

Did the study involve field work? ☐ Yes ☐ No

Field work, collection and transport

Field conditions	
Location	
Access & import/export	
Disturbance	

Reporting for specific materials, systems and methods

We require information from authors about some types of materials, experimental systems and methods used in many studies. Here, indicate whether each material, system or method listed is relevant to your study. If you are not sure if a list item applies to your research, read the appropriate section before selecting a response.

Materials & experimental systems

n/a	Involved in the study	
☐	☒	Antibodies
☒	☐	Eukaryotic cell lines
☒	☐	Palaeontology and archaeology
☒	☐	Animals and other organisms
☒	☐	Clinical data
☒	☐	Dual use research of concern
☒	☐	Plants

Methods

n/a	Involved in the study	
☒	☐	ChIP-seq
☒	☐	Flow cytometry
☒	☐	MRI-based neuroimaging

Antibodies

Antibodies used	Immunohistological staining: Dako-M0718 (CD68), Abcam-ab5694 (alpha-smooth muscle actin (αSMA)) and Dako-M0823 (CD31).
Validation	Experimental evidence on specificity is provided by the manufacturers on their website (Dako and Abcam)

Eukaryotic cell lines

Policy information about cell lines and Sex and Gender in Research

Cell line source(s)

Authentication

Mycoplasma contamination

Commonly misidentified lines
(See ICLAC register)

Palaeontology and Archaeology

Specimen provenance

Specimen deposition

Dating methods

☐ Tick this box to confirm that the raw and calibrated dates are available in the paper or in Supplementary Information.

Ethics oversight

Note that full information on the approval of the study protocol must also be provided in the manuscript.

Animals and other research organisms

Policy information about studies involving animals; ARRIVE guidelines recommended for reporting animal research, and Sex and Gender in Research

Laboratory animals

Wild animals

Reporting on sex

Field-collected samples

Ethics oversight

Note that full information on the approval of the study protocol must also be provided in the manuscript.

Clinical data

Policy information about clinical studies

All manuscripts should comply with the ICMJE guidelines for publication of clinical research and a completed CONSORT checklist must be included with all submissions.

Clinical trial registration

Study protocol

Data collection

Outcomes

Dual use research of concern

Policy information about dual use research of concern

Hazards

Could the accidental, deliberate or reckless misuse of agents or technologies generated in the work, or the application of information presented in the manuscript, pose a threat to:

No	Yes	
☒	☐	Public health
☒	☐	National security
☒	☐	Crops and/or livestock
☒	☐	Ecosystems
☒	☐	Any other significant area

Experiments of concern

Does the work involve any of these experiments of concern:

No	Yes	
☒	☐	Demonstrate how to render a vaccine ineffective
☒	☐	Confer resistance to therapeutically useful antibiotics or antiviral agents
☒	☐	Enhance the virulence of a pathogen or render a nonpathogen virulent
☒	☐	Increase transmissibility of a pathogen
☒	☐	Alter the host range of a pathogen
☒	☐	Enable evasion of diagnostic/detection modalities
☒	☐	Enable the weaponization of a biological agent or toxin
☒	☐	Any other potentially harmful combination of experiments and agents

Plants

Seed stocks

Novel plant genotypes

Authentication

ChIP-seq

Data deposition

☐ Confirm that both raw and final processed data have been deposited in a public database such as GEO.

☐ Confirm that you have deposited or provided access to graph files (e.g. BED files) for the called peaks.

Data access links
May remain private before publication.

Files in database submission

Genome browser session
(e.g. UCSC)

Methodology

Replicates

Sequencing depth

Antibodies

Peak calling parameters

Data quality

Software

Flow Cytometry

Plots

Confirm that:

☐ The axis labels state the marker and fluorochrome used (e.g. CD4-FITC).

☐ The axis scales are clearly visible. Include numbers along axes only for bottom left plot of group (a 'group' is an analysis of identical markers).

☐ All plots are contour plots with outliers or pseudocolor plots.

☐ A numerical value for number of cells or percentage (with statistics) is provided.

Methodology

Sample preparation	
Instrument	
Software	
Cell population abundance	
Gating strategy	

☐ Tick this box to confirm that a figure exemplifying the gating strategy is provided in the Supplementary Information.

Magnetic resonance imaging

Experimental design

Design type	
Design specifications	
Behavioral performance measures	

Imaging type(s)	
Field strength	
Sequence & imaging parameters	
Area of acquisition	

Diffusion MRI ☐ Used ☐ Not used

Preprocessing

Preprocessing software	
Normalization	
Normalization template	
Noise and artifact removal	
Volume censoring	

Statistical modeling & inference

Model type and settings	
Effect(s) tested	

Specify type of analysis: ☐ Whole brain ☐ ROI-based ☐ Both

Statistic type for inference

(See Eklund et al. 2016)

Correction

Models & analysis

n/a	Involved in the study
☐	☐ Functional and/or effective connectivity
☐	☐ Graph analysis
☐	☐ Multivariate modeling or predictive analysis

Functional and/or effective connectivity

Graph analysis

Multivariate modeling and predictive analysis

This checklist template is licensed under a Creative Commons Attribution 4.0 International License, which permits use, sharing, adaptation, distribution and reproduction in any medium or format, as long as you give appropriate credit to the original author(s) and the source, provide a link to the Creative Commons license, and indicate if changes were made. The images or other third party material in this article are included in the article's Creative Commons license, unless indicated otherwise in a credit line to the material. If material is not included in the article's Creative Commons license and your intended use is not permitted by statutory regulation or exceeds the permitted use, you will need to obtain permission directly from the copyright holder. To view a copy of this license, visit http://creativecommons.org/licenses/by/4.0/
